# A Case of Respiratory Epithelial Adenomatoid Hamartoma (REAH) in a Patient with History of Radiation Exposure

**DOI:** 10.1155/2019/9473608

**Published:** 2019-01-21

**Authors:** Darshan N. Patel, Yekaterina A. Koshkareva, Miles McFarland, Nathan A. Deckard

**Affiliations:** ^1^Resident Physician, Family and Community Medicine, University of New Mexico, Albuquerque, New Mexico, USA; ^2^Assistant Professor of Surgery, Cooper Medical School of Rowan University, Division of Otolaryngology, Cooper University Hospital, Camden, New Jersey, USA; ^3^Assistant Professor of Pathology, Cooper Medical School of Rowan University, Department of Pathology, Cooper University Hospital, Camden, New Jersey, USA; ^4^Assistant Professor of Surgery, Cooper Medical School of Rowan University, Director of Rhinology, ENT Allergy, and Skull Base Surgery Division of Otolaryngology, Cooper University Hospital, Camden, New Jersey, USA

## Abstract

Respiratory epithelial adenomatoid hamartomas (REAHs) are becoming a more commonly recognized otolaryngologic tumor and are often misdiagnosed as inverted papilloma. Here, we present such a case in a patient with history of previous radiation exposure. Otolaryngologists and pathologists should be aware of the mucinous histological appearance of REAH to help differentiate from other growths. Given our patient's history, an association between REAH and previous radiation exposure is worth consideration.

## 1. Introduction

Respiratory epithelial adenomatoid hamartomas (REAHs) are becoming a more commonly recognized otolaryngologic tumor. REAHs are often misdiagnosed as inverted papilloma, with accurate diagnosis limited by a low index of suspicion. While the malignant potential of inverted papilloma should not be underestimated, REAHs may require less extensive management. Thus, an accurate diagnosis is important in preventing unnecessary intervention.

## 2. Case Report

Case reports are exempt from institutional review board approval at our institution.

A 75-year-old woman was evaluated for long standing right-sided nasal obstruction, dependent mouth breathing, clear rhinorrhea, congestion, and hyposmia not relieved by intranasal steroids or nasal irrigations. She initially presented with a history of previous endoscopic sinus surgery in Russia more than 20 years ago followed by nasal polyposis treated with ambulatory cauterization. She was also noted to have a history of nonmelanotic skin cancers of the nose treated with radiation in Russia.

**Figure 1:**
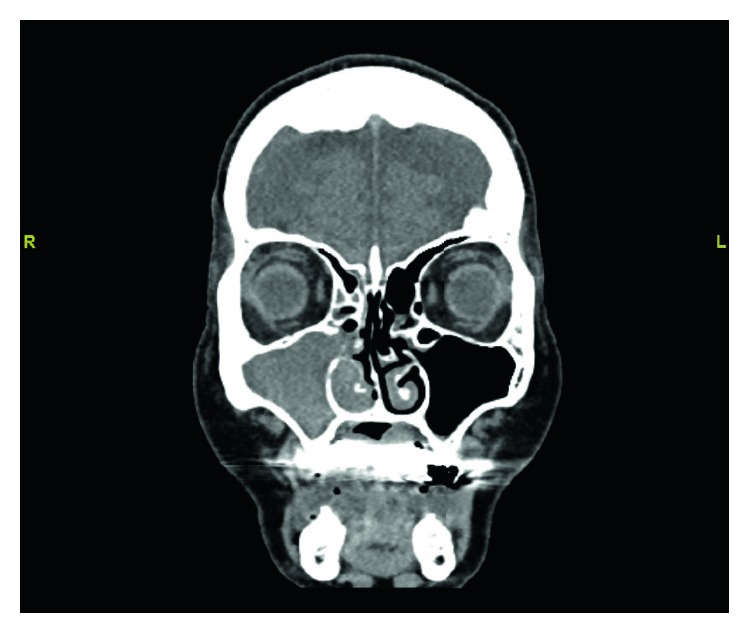
Coronal CT scan, soft tissue window, showing complete opacification of the right maxillary sinus, obstruction of the right ostiomeatal complex, and soft tissue density in the right inferior meatus.

Physical exam findings included a fleshy intranasal lesion that, in the setting of previous nasal skin cancer treated with radiation, raised a concern for possible secondary carcinoma. Computed tomography showed complete opacification of the right maxillary sinus, obstruction of the right ostiomeatal complex, and soft tissue density in the right nasal passage (Figure 1).

**Figure 2:**
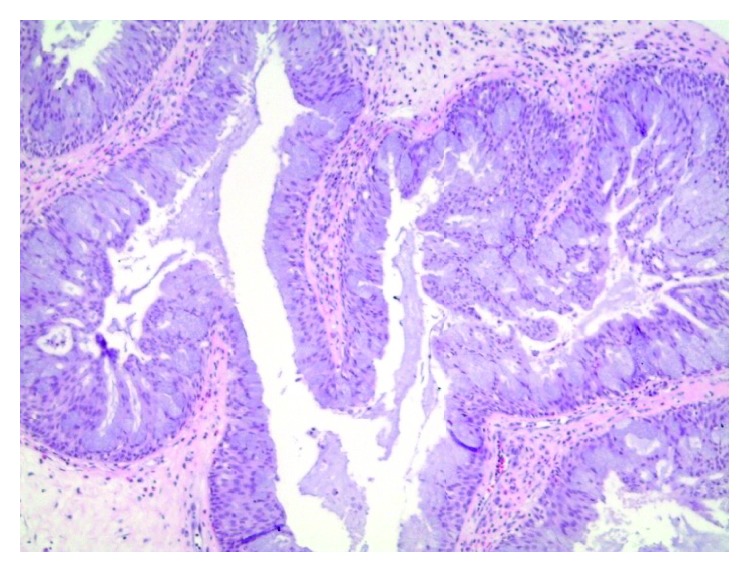
Right nasal lesion. Hematoxylin and eosin stain. Much of this polypoid tumor had areas lined by mucin-containing cells (100x).

Initial biopsy revealed a nasal mass that originated in the right inferior meatus. Pathology showed multiple polypoid fragments lined by a respiratory type epithelium with underlying edematous stroma with mild chronic inflammation. There was invagination of the surface epithelium into the underlying stroma resulting in nested aggregates of bland glandular and mucinous cells and focally benign squamous epithelium (Figure 2). These features were found to be consistent with a benign inverted papilloma.

The patient presented with continued nasal obstruction and was evaluated for definitive treatment. Given her diagnosis of inverted papilloma and chronic rhinosinusitis, complete excision and revision endoscopic sinus surgery was recommended. During endoscopic sinus surgery, an exophytic mass with abnormal maxillary mucosa was seen emanating from the left inferior meatus that was thought to originate from the right maxillary sinus, given that it was protruding through a bony dehiscence into the inferior meatus and nasal cavity. Right partial inferior turbinectomy was performed, along with right extended maxillary antrostomy and stripping of maxillary mucosa to remove the entirety of the presumed inverted papilloma base within the maxillary sinus. Intraoperative and postoperative pathology again showed inverted papilloma.

**Figure 3:**
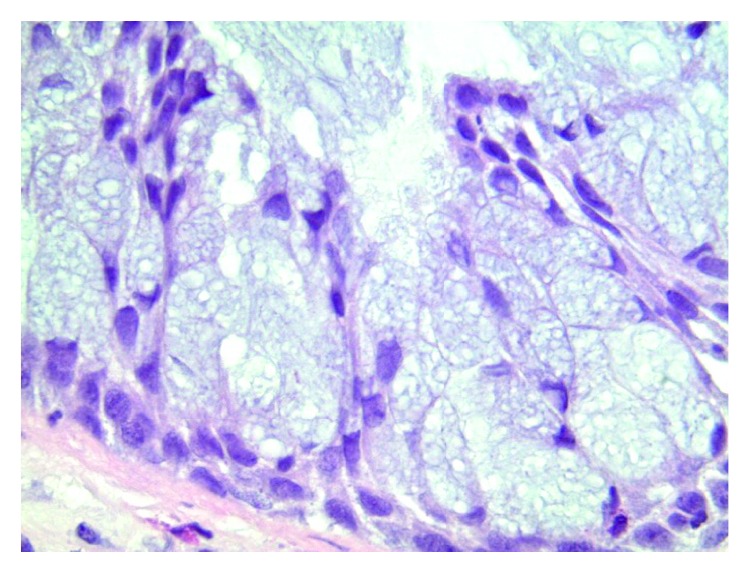
Right inferior meatus mass. Hematoxylin and eosin stain. Gland-like structure lined by one or more layers of cells, with mucinous, vacuolated cells predominating towards the lumen. The nuclei are bland (630x).

Given her suspicious history, a second opinion was requested. This noted stromal hyalinization characterized by thickened, eosinophilic basement membrane-like material. The polypoid lesion had an inverted growth pattern concerning for inverted papilloma, but it had a prominent glandular lining (Figure 3), leading to a revised diagnosis of REAH.

## 3. Discussion

Many REAHs are found in the olfactory groove and associated with olfactory groove widening [1, 2]. Other common locations include the nasal septum, lateral nasal wall, sinus cavities, and olfactory cleft [3]. Previous reports have established an association between REAH and nasal polyposis [3–5]. The average age at which REAH diagnosis occurs is 54 years old, with a 3 : 2 male-to-female predominance [1].

Typical presenting symptoms include the following: nasal obstruction, nasal stuffiness, deviated septum, epistaxis, and chronic rhinosinusitis occurring from a few months to many years in duration [6]. Differential diagnosis for REAH can include the following: inflammatory polyps, inverted papilloma, and low-grade adenocarcinoma [7]. The only definitive diagnosis is biopsy, with histology characteristics of prominent glandular proliferation lined by ciliated respiratory epithelium originating from the surface epithelium [6]. This should not be confused with the inverted growth pattern of inverted papilloma, which originates from invagination of hyperplastic squamous and/or respiratory epithelium. Furthermore, the basement membrane around epithelial islands is thin in inverted papilloma in contrast to the thick, hyalinized basement membrane of REAHs [7].

The etiology of REAH is unclear, with no link to any specific exposures. That said, it is interesting to note that our patient had previous radiation exposure in the same region as her eventual REAH presentation. REAHs are typically associated with a benign course with no reported cases of recurrence after complete surgical resection [8].

Greater recognition of REAHs as distinct from inverted papilloma, inflammatory polyps, and low-grade adenocarcinoma can help determine appropriate management and treatment. As REAHs are often benign and self-limiting, they do not need to be resected in asymptomatic patients or as aggressively resected in symptomatic patients. Patients should be followed with serial endoscopies to evaluate for possible disease progression, and conservative management may be sufficient.

In conclusion, REAH is becoming a more widely recognized tumor. Expertize in pathology is required for diagnosis, and conservative management is the current treatment approach in asymptomatic patients. Otolaryngologists and pathologists should be aware of the mucinous histological appearance of REAH to help differentiate from other growths. In our patient, the disease was focused in the inferior meatus. Our findings support surveillance and diagnosis of REAH in other locations. Given our patient's history, an association between REAH and previous radiation exposure is worth consideration.
